# Community intervention strategies to reduce the impact of financial strain and promote financial well-being: a comprehensive rapid review

**DOI:** 10.1177/1757975920984182

**Published:** 2021-02-18

**Authors:** Nicole M. Glenn, Lisa Allen Scott, Teree Hokanson, Karla Gustafson, Melissa A. Stoops, Brynn Day, Candace I. J. Nykiforuk

**Affiliations:** 1 Centre for Healthy Communities, School of Public Health, University of Alberta, Edmonton, Alberta, Canada; 2 Alberta Cancer Prevention Legacy Fund, Population, Public and Indigenous Health, Alberta Health Services, Calgary, Alberta, Canada; 3 Department of Community Health Sciences, Cumming School of Medicine, University of Calgary, Calgary, Alberta, Canada; 4 Population, Public and Indigenous Health, Alberta Health Services, Calgary, Alberta, Canada; 5 Policy, Location and Access in Community Environments Research Lab, School of Public Health, University of Alberta, 3-300 Edmonton Clinic Health Academy, Edmonton, Alberta, Canada

**Keywords:** rapid review, financial strain, financial stress, financial well-being, community interventions, community strategies, interviews, environmental scan

## Abstract

Financial well-being describes when people feel able to meet their financial obligations, feel financially secure and are able to make choices that benefit their quality of life. Financial strain occurs when people are unable to pay their bills, feel stressed about money and experience negative impacts on their quality of life and health. In the face of the global economic repercussions of the COVID-19 pandemic, community-led approaches are required to address the setting-specific needs of residents and reduce the adverse impacts of widespread financial strain. To encourage evidence-informed best practices, a provincial health authority and community-engaged research centre collaborated to conduct a rapid review. We augmented the rapid review with an environmental scan and interviews. Our data focused on Western Canada and was collected prior to the pandemic (May–September 2019). We identified eight categories of community-led strategies to promote financial well-being: systems navigation and access; financial literacy and skills; emergency financial assistance; asset building; events and attractions; employment and educational support; transportation; and housing. We noted significant gaps in the evidence, including methodological limitations of the included studies (e.g. generalisability, small sample size), a lack of reporting on the mechanisms leading to the outcomes and evaluation of long-term impacts, sparse practice-based data on evaluation methods and outcomes, and limited intervention details in the published literature. Critically, few of the included interventions specifically targeted financial strain and/or well-being. We discuss the implications of these gaps in addition to possibilities and priorities for future research and practice. We also consider the results in relation to the COVID-19 pandemic and its economic consequences.

## Introduction

The novel coronavirus disease (COVID-19) will have long-lasting repercussions on people’s health and well-being worldwide. Beyond immediate health consequences, there will be serious physical, social and mental health impacts of added financial strain (1). Prior to the current economic crisis, people in Canada (2) and globally (3) were feeling the impacts of financial strain. These numbers have since risen (4), with job loss and the collapse of financial markets worldwide (5). In the years ahead, community-led interventions will be required to help fill the gaps left by broad policy actions to address financial well-being, and tailor to the unique needs of specific populations and settings (6). This is particularly vital for underserved groups (6) who are most likely to experience unjust and avoidable differences in financial strain (1,7). This article reports on findings from a rapid review of community interventions to address financial strain and promote financial well-being. To align with the needs of our practice partners on the project, the Public, Population and Indigenous Health Strategic Clinical Network, Alberta Health Services, we operationalised community as a geographic jurisdiction, such as a city, town, neighbourhood or region (in the case of rural locations) and/or people or groups of people living within those geographical limitations.

Financial strain occurs when people cannot pay their bills, feel worried about money and experience negative impacts on their quality of life and health (8,9). Financial strain is a subjective measure of current circumstances (8,9). In contrast, financial well-being includes the present and future as well as subjective and objective measures (9,10). It is the perception of one’s ability to sustain a current desired living standard and financial freedom (10,11). Financial strain is an aspect of financial well-being (9,10,12,13). It differs from objective measures of financial circumstances (e.g. poverty, employment status, income) (1), which have well-documented relationships with health (14). Independent of objective measures of financial circumstances (e.g. income), financial strain has demonstrated negative impacts on people’s health (15,16) that are worthy of consideration.

Financial strain disproportionately impacts underserved groups (e.g. Indigenous peoples, racialised peoples) (1,7) who experience systemic barriers that result in inequities in health, well-being, employment and education outcomes (17). People who have greater privilege, own a house and have a stable income can also experience financial strain (18). Financial strain can shift over the life-course (19) and due to unexpected events (e.g. illness, job loss; 20). The broader socio-economic environment also shapes financial strain (8,19) through complex, inter-related factors such as the societal and economic impacts of COVID-19 (5,21).

Financial strain and well-being and their connection to health are emerging research areas (1,9,10). Financial strain increases the risk of depression, anxiety, marital problems, health-damaging behaviours, poor physical health and mortality (20,22–25). Children of people who experience financial strain have elevated risk of mental illness, disability and loneliness (22,26). However, how to address financial strain and promote financial well-being, particularly by community-level actors and organisations, is not well-understood (1). Drawing on data collected through a rapid review, environmental scan and organisational interviews, we identified eight community-led intervention strategies to promote financial well-being. We describe them and reflect on their potential to support the health and well-being of communities facing the repercussions of the pandemic. We identify gaps in understanding and offer recommendations for future investigations and interventions.

## Methods

This project was a collaboration between practice partners at the Public, Population and Indigenous Health Strategic Clinical Network, Alberta Health Services and researchers at the Centre for Healthy Communities, School of Public Health, University of Alberta. The project received ethical approval from the Human Research Ethics Board, University of Alberta (reference number: Pro00090909). Our primary objective was to create a toolkit for use by community organisations and municipal/regional governments with implementation details for interventions to promote financial well-being (https://together4health.albertahealthservices.ca/FinancialWellness/news_feed/building-financial-well-being-a-community-planning-toolkit). To achieve this, we conducted a rapid review of the academic literature (27). We supplemented this with an environmental scan of websites for Canadian community-led interventions and interviews with Alberta-based organisations intervening to address financial strain and/or well-being in their communities. What resulted was a six-step process.

### Step 1: Research/practice question development

We used the PICO (Population, Intervention, Comparator, Outcome; 27) framework to develop our research/practice question: what interventions can communities use to address financial strain and promote financial well-being among local residents?

### Step 2: Search strategy

A Masters in Library and Information Science (MLIS)-trained Information Specialist helped to develop the search terms and conducted the academic literature searches using two online databases (Ovid PsycINFO, Social Sciences Index) as well as Google and Google Scholar (on 11 May 2019). For the environmental scan, she created 15 distinct search strategies that were run by research assistants in Google (9–21 May 2019, 19 June 2019 and 19 July 2020). The searches included terminology related to three concepts: (a) financial strain and/or well-being; (b) intervention; and (c) community.

### Step 3: Source appraisal

A single reviewer used a two-step screening process to identify relevant articles for the rapid review. Inclusion criteria were: community-level interventions targeting financial well-being and/or strain; process or outcome studies that had been evaluated; primary research studies, reviews, practice-based reports; published in English or French between 2014 and 2019; United Nations (UN) developed economy (28). Primary screening involved title and abstract review. Secondary screening involved a full-text review of all articles that passed primary screening. We searched the reference lists of included articles for additional relevant articles. We used the Scopus search engine to identify articles that cited the included articles. A total of 21 articles were included in the rapid review.

The environmental scan searches were run, and the first 200 records were screened by two independent reviewers. Websites that contained additional relevant links, such as Alberta-based municipal websites, were ‘hand-searched’ by following embedded links and applying inclusion criteria. Inclusion criteria were: Canadian interventions targeting financial well-being and/or strain among families, individuals or communities; English or French. A reviewer bilingual in French and English conducted all of the French language searches, screening and data extraction. A second reviewer conducted the English language searches, screening and data extraction.

A total of 194 unique interventions emerged from the scan. We conducted full data extraction on 7 Alberta-based poverty reduction strategies and 96 Alberta-based community-level interventions. The remaining 91 interventions were national/provincial-level or community-level from outside Alberta, which underwent abbreviated data extraction and did not inform the findings of this rapid review.

### Step 4: Interviews

Our interview guide focused on gaps in our rapid review and environmental scan results, and gathered information on intervention implementation and tailoring by setting or population. We identified interview participants through the environmental scan, prioritising Alberta-based organisations running unique interventions or operating outside of urban centres. A total of 12 semi-structured telephone interviews occurred with 15 participants between 3 September and 17 September 2019. These represented 12 distinct interventions and 10 organisations (29–39). Additional interview details are available in a forthcoming publication. Interviews were recorded and transcribed professionally. All participants provided informed consent. Interviews averaged 68 min (range: 51–95 min).

### Step 5: Evidence synthesis

A single reviewer extracted (i.e. coded) detailed content from the articles, websites and interview transcripts using NVivo software (40). This included identifying information, population and setting details, study details, outcomes and/or process evaluations, limitations, implementation details and lessons learned. We used an inductive-deductive, line-by-line approach to content analysis (41) for the analysis. The coding scheme was developed based on the transcripts, interviewer notes and findings from the environmental scan and rapid review.

We first synthesised the findings from the rapid review and environmental scan to identify specific intervention strategies (e.g. housing) and sub-strategies (housing policies). Our analysis of the interview transcripts focused on additional details (e.g. tips for tailoring) relevant to the intervention (sub-)strategies identified. It also involved pinpointing new sub-strategies that emerged from these conversations (e.g. alternative transportation).

### Step 6: Identification of key practice implications and gaps

Two co-authors reviewed the data extracted and identified key practice implications and gaps in the evidence that aligned with each of the eight intervention strategy areas. These were then discussed with the full team until we reached a consensus.

### Methodological limitations

Our study results are contextualised by our methodological limitations. To maintain the narrow scope required of a rapid review, and to prioritise the literature most relevant to the needs of our practice partners, articles that only described interventions, reported on study protocols or discussed theoretical or predictive models in the absence of intervention-related outcomes or process measures were excluded. These may have contained detailed implementation information; therefore, their exclusion represents a possible limitation of the rapid review method. Limiting academic articles to UN developed economies (28), the environmental scan to Canadian websites and the in-depth extraction for the environmental scan to Alberta-based organisations/interventions and poverty reduction strategies may have also excluded potentially relevant interventions (e.g. Prosper Canada’s financial empowerment interventions; 42). Due to the rapid nature of this project, we did not contact organisations/interventions found through the environmental scan, beyond those who participated in interviews, to obtain more detailed information (e.g. evaluation reports). We also followed a structured search for the environmental scan (i.e. using a search string), which meant relevant organisations/interventions could have been missed. Finally, the sample size for our interviews was small and all organisations were Alberta-based. Nevertheless, the diversity of the Alberta context along with our comprehensive approach that targeted interventions/organisations in varied settings for different populations (e.g. Indigenous peoples, seniors, single-parent families) suggest that our findings may be broadly applicable to people and communities in similar jurisdictions who experience financial strain.

## Results

### Evidence summary

Based on the rapid review and environmental scan, we identified eight distinct intervention strategies for communities to act on financial well-being and/or financial strain. They included: (a) systems navigation and access; (b) financial literacy and skills; (c) emergency financial assistance; (d) asset building; (e) events and attractions; (f) employment and educational support; (g) affordable and accessible transportation; and (h) housing. We noted intervention sub-strategies for six of the eight strategies, which are detailed along with a description of the intervention strategies, related target populations and outcomes in Supplementary Table S1.

Of the 21 articles retrieved through the rapid review, 11 reported on multi-component interventions (i.e. they included two or more of the intervention strategies; 43–48) or on interventions with wrap-around services and support (e.g. childcare, healthcare; 49–53). This was also the case of the interventions included within the environmental scan and discussed in the interviews. All but one of the interventions included in the rapid review (54) targeted specific underserved populations. Underserved populations, particularly low-income people and families, people experiencing poverty, youth, seniors and un- or under-employed people were also the frequent target of the interventions/organisations included in the environmental scan. Nearly all of the articles included in the rapid review focused on interventions that took place in large urban population centres in the Unites States (US) (43–45,47,49–51,54–63). One article reported on a Canadian intervention (46) and another on an Australian intervention (52), both in large urban population centres.

Overall, the rapid review, environmental scan and interview evidence that reported on outcomes found improvements in measures of financial well-being (e.g. savings, income, reaching financial goals; 29–33,47,49–51,54–56,61) and financial behaviours and/or skills (e.g. money management; 29,32,34,35,47–51,54,59,62). They also reported benefits related to mental, physical and social health (e.g. depression, social connection, self-esteem, personal safety; 30,36,37,44,45,47,50,56,57,59,61,64,65) healthy lifestyle behaviours (e.g. preventative healthcare, fast food consumption; 30,49,51,56,58,59,61), motivation to change (43,51), crime recidivism (30,34,38), and positive educational (e.g. school attendance; 45,51,55) and employment skills and achievements (e.g. employment rate, training certificates; 30,33,38,45,49,51,52). People who have received support, for example, one-time emergency funding, reported donating to the program at a later date (39).

Not all interventions had the intended effect. For example, the participants in a matched savings program who had a very low income increased their motivation to save but found it difficult to actually save due to limited income and pressing financial demands (43). Although credit card debt was reduced, overall debt increased (due mostly to student loans) among single mothers participating in a comprehensive educational support program (51). One primary healthcare-based intervention found that, although the program improved money management skills and confidence, it also resulted in increased resistance to new activities among participants and stress when talking to staff about finances (48). According to those we interviewed, people whose application for emergency funding is not accepted, for example, may be upset that they were not approved (32,39). Interviewees also pointed out that even with reduced fees for local events and attractions, some people will not be able to participate due to the costs involved (35). Some interviewees cautioned that the services provided by non-profits and other non-governmental organisations were often ‘band-aids’ and did not address systemic underlying causes of financial strain (32). In essence, the existence of community-led interventions (e.g. services, programs) enabled governments to provide insufficient basic needs support (i.e. income benefits, affordable housing).

### Evidence gaps and limitations

The evidence reviewed was limited in relation to understanding intervention outcomes and impacts. Specific outcomes attributable to the individual intervention (sub-)strategies was lacking because the majority of the interventions assessed in the rapid review were multi-component or included wrap-around support (43–53), were focused on process measures rather than outcomes (46,57), and/or according to the article authors had limited generalisability (44,45,47,49,50,53,54–56,58,59,61–63) and/or significant methodological limitations (e.g. small sample size, use self-report data; 43,46,52,53,56,59,61,63). The environmental scan data were restricted due to lack of details provided on evaluation methods and outcomes. Interviewees were asked about intervention evaluation results and methods. Nevertheless, most of the interventions had not been assessed formally and/or the evaluation information that was available was minimal or anecdotal (e.g. ‘change stories’). Many of the people we interviewed discussed the challenges of conducting effective evaluations of interventions, which we have reported in a forthcoming publication of the results of the interviews on how communities can lead interventions to address financial well-being and/or strain (manuscript submitted). Due to a lack of outcome/impact details available (except: 65–67), the environmental scan contributed minimally to the results of the study beyond identifying potential intervention strategies, populations and settings as well as interview candidates.

Few research articles reported on follow-up assessments to evaluate the sustained impacts of the interventions. The results of these assessments were mixed. In one case, the effects mostly dissipated (56), while in another they were maintained (61). Health and lifestyle related benefits (e.g. changes in health behaviours, such as fast food consumption and exercise) of interventions focused on financial well-being may take longer than financial benefits (i.e. reduced spending and financial stress; increased savings and self-control) to change significantly, therefore longer-term follow ups (i.e. >1 year) may be required to see these changes (59).

Although interventions commonly targeted social determinants of health (e.g. education, income; 44,45,48,50,51,55), they focused on individual and family-level change (e.g. matched savings, behavioural change) and did not address higher-level (i.e. community, society) causes of financial strain or well-being (e.g. access to healthcare). With a few exceptions (47,50,51), the evidence that focussed on higher levels of change, such as transportation, housing, and access to banking came from the practice interviews and environmental scan and, therefore, was lacking detailed outcome or impact information.

Limited intervention details were provided within the rapid review articles. They focused on methodological descriptions and outcome reporting. Beyond a few research articles (43,59–61,63), study authors did not discuss how to tailor interventions to setting or population. Fidelity to the intervention was also rarely assessed in the literature/evidence reviewed. However, a study of the relationship between intervention implementation fidelity and outcomes found it was key to success (52).

Except for two articles (49,63), the interventions included in the rapid review did not target financial well-being and/or strain specifically. They had adjacent foci, such as economic stability or security (43,46,48,51,62), economic self-sufficiency (44), financial capability (47,57), financial hardship (55,56,58), financial stress (59–61), employment (51–52), poverty reduction (43,50,55,56,58), and changes in financial behaviours and skills (54,59–61). Many interventions also had improved health and well-being as an objective (45,48,55,56,58).

## Discussion

Research on how to best promote financial well-being or address financial strain at the community-level is in its infancy (1,10). The results of our investigation revealed that there is little evidence to support specific recommendations for best practices in this area. Published research that describes the outcomes of financial strain and/or well-being interventions, their mechanisms for addressing financial well-being or strain, for whom and under what circumstances, is lacking. Practice-based evidence contains vital implementation details (e.g. how to tailor to populations and settings); however, detailed intervention descriptions, evaluation results and methods, and tips or lessons learned are difficult to access and/or unavailable. Making this information more readily available to researchers and other organisations working in practice would help to address the gap we noted from the practice-based literature. Additionally, the use of a realist lens for intervention evaluations and/or the reporting of contextual details and mechanisms (i.e. program resources that influence actions) along with outcomes could provide a deeper understanding of which interventions would best be implemented by specific organisations for particular contexts/populations to achieve desired outcomes (68).

In this pandemic, community-led initiatives, such as the eight intervention strategies outlined herein, will be increasingly vital to ease the impact of the financial strain and promote financial well-being in ways that are meaningful to local populations and settings (7,69). Community-led, bottom-up approaches are key to effective health promotion, particularly for underserved populations (69), who are disproportionately impacted by financial strain (5,21). Community organisations, non-governmental agencies and not-for profits acting at the community-level, and municipal/regional governments are well-positioned to assess and address the needs of the local population in ways that align with contextual constraints and opportunities and are asset-based (69). That said, to address the root causes of financial strain and promote financial well-being in the longer-term will require substantive action at higher levels of governments on the social determinants of health (14). For example, strengthening employment insurance, health care, childcare (and other caregiving), and affordable housing policies could ‘bend the curve’ of societal impacts resulting from increased financial strain experienced by communities and individuals (70). Targeted research on the impact of social policies on financial strain and/or well-being among diverse populations will be required to direct equitable government action in the area (70). There is currently a unique opportunity to undertake such investigations of the diverse policies introduced by governments across the globe to address the economic fallout of the pandemic (e.g. 71,72).

How to best align bottom-up and top-down strategies to address financial strain and promote financial well-being in the aftermath of the pandemic will require serious consideration. Assessing the impact of interventions that have arisen in response to COVID-19 (e.g. emergency housing, income and employment policies; 71,72) and how changes brought about by the pandemic have affected the outcomes of existing community-led interventions (e.g. the move to online delivery, reduced budgets and staff) will be key to success. Considering the impacts specifically on underserved populations will be required to ensure equitable outcomes. More robust, disaggregated data that includes information on race, gender, immigration status and socio-economic status among other social categories is necessary to understand the short- and long-term impacts of these financial well-being intervention strategies and their equity implications – that is, what works for whom, where, and how.

### How can communities support financial well-being?

The results of this rapid review have revealed that the evidence-base for community-led interventions to promote financial well-being and address financial stain is limited. The gaps in the evidence notwithstanding, people are facing unprecedented financial shocks due to repercussions of COVID-19. Communities and governments have already begun to respond (e.g. emergency income and employment policies; 71,72), and will need to continue to do so over the coming years. Given the urgent nature of situation, we recommend that community-led strategies to address financial strain and promote financial well-being include the following:

- Wrap-around support (e.g. healthcare) or connections to this support if it is required. This includes the provision of basic needs (e.g. food, shelter), which is particularly key for underserved populations and people living outside of resource-dense urban centres.
- Robust evaluations, that collect information (e.g. data, stories) on the needs, processes, outcomes (social, physical and mental health) and impacts of interventions implemented. Reporting data on socially ascribed categories (e.g. race, ethnicity, socio-economic status) will be key to understanding what works for whom and how it does so, the possible unintended consequences of the interventions and also equity impacts. A plan for sharing lessons learned and evaluation findings with others working in practice and researchers [is necessary] to inform the creation of best practices.

Financial strain is a socio-economic pandemic that will echo each wave of COVID-19. Promoting financial well-being at this time necessitates adequate preparation and swift, strategic community-led action to support societal recovery, particularly for underserved populations who are already at heightened risk of the negative impacts of both pandemics.

## Supplemental Material

sj-pdf-1-ped-10.1177_1757975920984182 – Supplemental material for Community intervention strategies to reduce the impact of financial strain and promote financial well-being: a comprehensive rapid review Click here for additional data file. Supplemental material, sj-pdf-1-ped-10.1177_1757975920984182 for Community intervention strategies to reduce the impact of financial strain and promote financial well-being: a comprehensive rapid review by Nicole M. Glenn, Lisa Allen Scott, Teree Hokanson, Karla Gustafson, Melissa A. Stoops, Brynn Day and Candace I. J. Nykiforuk in Global Health Promotion
