# Supplementary material for: Community intervention strategies to reduce the impact of financial strain and promote financial well-being: a comprehensive rapid review
Source: Glob Health Promot. 2021 Feb 18;28(1):42–50. doi: 10.1177/1757975920984182 (PMC7897542; doi:10.1177/1757975920984182)
Supplement: sj-pdf-1-ped-10.1177_1757975920984182 – Supplemental material for Community intervention strategies to reduce the impact of financial strain and promote financial well-being: a comprehensive rapid review [file sj-pdf-1-ped-10.1177_1757975920984182.pdf]

Online Only Supplementary Table S1. Summary of Community Intervention Strategies to Promote Financial Well-Being

Intervention (Sub-)Strategy and Description	Target Population (s)	Outcomes and/or Impacts*	Evidence (Citation #)†
**1. Systems Navigation and Access**			
**Systems Navigation** Connect people to available services and benefits. Assist with applications. Provide community outreach.	People in need of assistance; vulnerable and/or low-income parents; 'pre-seniors'; people experiencing or at risk of homelessness; primary health care patients; families part of family and maternal health program; single mothers	• Improved financial well-being, financial skills, sense of community, confidence about financial skills, sense of accountability, coping skills, motivation to change, savings, optimism, awareness and reach of programs and services, meeting of financial goals, housing security, and employment.<br>• Increased sense of support, sense of hope, ability to cope with life's difficulties, and income. Reduced debt / burden.	31, 36, 37, 45-47, 49, 50, 52
**Free Tax Clinics** i.e., Canada Revenue Agency volunteer tax program.	People with a modest- or low-income	• Brings money back into families and the community.<br>• Improved financial well-being, sense of support, behaviours, and skills. Reduced stress and frustration.	32, 35
**Accessible Banking** Low-barrier banking services in partnership with a banking institution.	Underserved populations; people with barriers to banking (e.g., homelessness, addictions); women and youth with a very low income	• Improved financial well-being and skills, savings, ability to make money last, meeting of financial goals, housing security, employment, confidence with savings, budgeting, spending, financial self-efficacy, attitudes, positive financial behaviours, and personal safety among customers.	29, 44, 48, 50
**Identification Services** Assistance getting and storing government identification.	Underserved populations; people with extreme barriers to gaining identification (e.g., homelessness, addictions)	• Facilitated access to other benefits and services, such as banking. Increased safety.	29

[*] Due to limitations in study design and measurement tools, causality and/or the connection between outcomes and (sub-)strategies cannot be determined. The outcomes/impacts listed are only possible and can vary in relation to the intervention strategies, implementation, setting, participants, and other contextual factors.
[†] Limited to evidence that reported on outcomes and/or impacts.

Intervention (Sub-)Strategy and Description	Target Population (s)	Outcomes and/or Impacts*	Evidence (Citation #)†
**2. Financial Literacy and Skills**			
**Financial Literacy**<br>Teach and build financial skills (e.g., budgeting, asset-building).	People with a very low income, experiencing poverty, precariously housed, and/or facing complex challenges; vulnerable parents; people experiencing or at risk of homelessness; primary health care patients; youth with a low income; families part of family and maternal health program; employees	• Increased financial wellness, knowledge, skills, quality of life, and hope. Improved financial well-being, behaviours, skills, savings, optimism, healthy behaviours, feelings of support and safety to discuss financial issues.<br>• Reduced stress, financial stress, use of payday loans, overdue bills, and frustration. Facilitated the meeting of financial goals and securement of housing and employment.<br>• Unintended negative consequences: unpredictable income and fear of not qualifying for benefits can be a barrier to saving. Can lead to increased stress around discussing financial issues with program staff and reluctance to try new activities.	32, 34, 44, 47-51, 55, 58, 60-62, 64
**Financial Coaching**<br>Personalized financial guidance and mentorship.	People with a very low income, experiencing poverty, precariously housed, and/or facing complex challenges; people experiencing or at risk of homelessness; single mothers; primary health care patients	• Improved financial well-being, behaviours, skills, savings, goal-setting, self-esteem, quality of life, hope, confidence, parenting skills, motivation to change, coping skills, income, optimism, healthy behaviours, and financial management. Increased feelings of support and safety to discuss financial issues.<br>• Reduced stress, debt, financial stress, use of payday loans, overdue bills, and frustration. Facilitated the meeting of financial goals and securement of housing and employment.<br>• Unintended negative consequences: unpredictable income and fear of not qualifying for benefits made it difficult for some participants to change behaviours related to savings.	32, 45, 47, 50-52, 58, 60- 62

Intervention (Sub-)Strategy and Description	Target Population (s)	Outcomes and/or Impacts*	Evidence (Citation #)†
**3. Emergency Financial Assistance**			
**Emergency funds.** Often available only one-time. Can take the form of grocery or other gift cards.	People in need of financial assistance; residents with a low income; people experiencing disability; parents; seniors	• Positive impact on financial well-being. Improved sense of community. Helps people facing financial challenges.<br>• Unintended negative consequence: some people feel upset if they do not receive the funding.	31, 32, 35, 39
**4. Asset Building**			
**Matched Savings** Cash incentive for personal savings.	People with a very low income, experiencing poverty, precariously housed, and/or facing complex challenges; people experiencing or at risk of homelessness; single parent mothers; youth with a low income; people using free tax clinics	• Improved financial well-being, behaviours, skills, optimism, and savings. Reduced stress and frustration.<br>• Facilitated the meeting of financial goals and securement of housing and employment.	32, 44, 48, 50, 51, 63
**Conditional Cash Incentives** Cash incentive for specific behaviours or actions.	Parents with a low-income	• Increased savings for children's future, spending on children, educational attainment, employment in the sector where they trained, optimism, and self-efficacy.<br>• Children increased time spent on schooling and academic activities.	46, 56, 57, 59

Intervention (Sub-)Strategy and Description	Target Population (s)	Outcomes and/or Impacts*	Evidence (Citation #)†
**5. Events and Attractions**			
Low-cost or free local access to cultural, recreation, and sporting events, attractions, and programs.	Underserved populations; residents with a low income; people in need of assistance; people experiencing disability; parents; seniors	• People feel better able to get by financially on their monthly income and reconnect or form relationships in their community.<br>• Improved community participation and sense of belonging.	33, 35
**6. Employment and Education Support**			
**Employment Support**<br>Job training, career planning, skill building, and opportunities.	People with barriers to employment; people experiencing or at risk for homelessness, mental illness, and/or addictions; youth with a low income; single mothers	• Improved employment, employability, financial well-being and skills, motivation to change, income, savings, optimism, and coping skills.<br>• Some people have experienced improvements in housing stability/circumstances and addictions.<br>• Facilitated the meeting of financial goals and securement of housing and employment. Reduced debt.	34, 38, 48, 50, 52-54
**Educational Support**<br>Wrap-around support to enable education pursuit and employment attainment.	People with a low income; underserved populations; women experiencing complex challenges; single-parent families; Indigenous youth; single mothers	• Increased educational attainment and employment in the sector they trained for, improved employment skills, savings, income, confidence, motivation to change, coping skills, optimism, self-efficacy, sense of security, personal growth and learning, particularly about financial management.<br>• Reduced debt, stress, and crime recidivism.	34, 46, 51, 52
**Social Enterprise**<br>Employment and community-building business.	People with barriers to employment; people experiencing homelessness, mental illness, and/or addictions	• Improved visibility within the community, employment and employability, getting people off the streets, housing stability, and addictions.<br>• Increased knowledge about the services/programs.	38

Intervention (Sub-)Strategy and Description	Target Population (s)	Outcomes and/or Impacts*	Evidence (Citation #)†
**7. Transportation**			
**Public Transportation**<br>Free or discounted access to public transportation.	People with a low income; underserved populations; youth; seniors; Indigenous youth/adults/women; women experiencing poverty	• Outcomes/impacts were not available.	34, 35, 67, 68
**Taxi Service**<br>Subsidized local or regional taxi service.	Seniors and adults with permanent disabilities for subsidized transportation; and those in need of assistance	• People reported satisfaction with the service.	35
**Alternative Transportation**<br>Affordable alternatives to public transportation.	People in need of assistance; seniors and adults with permanent disabilities for subsidized transportation	• Positive impact on financial well-being.<br>• Improved sense of community.	31, 35
**8. Housing**			
**Rental subsidies and supplements**<br>Subsidized rental units, social housing, or supplements to cover rent.	People with low income, minimally or part-time employed or receiving income benefits, homeless, or at-risk of homelessness; single parent mothers	• Improved housing stability, employment and related skills, health, and healthy behaviours.<br>• Reduced stress, increased savings, feelings of security, homelessness.<br>• People have paid off 'street debt' and other loans (e.g., payday lenders).<br>• Housing choice has greatly reduced re-housing.	30, 51, 66

Intervention (Sub-)Strategy and Description	Target Population (s)	Outcomes and/or Impacts*	Evidence (Citation #)†
**Supportive housing**<br>Housing with medical and/or social support.	People with low income, minimally or part-time employed or receiving income benefits, homeless, or at-risk of homelessness	• Improved housing stability, employment and related skills, health, and healthy behaviours.<br>• Reduced stress, increased savings, feelings of security, homelessness.<br>• People have paid off 'street debt' and other loans (e.g., payday lenders).<br>• Housing choice has greatly reduced re-housing.	30
**Housing policies**<br>Regulations and fiscal incentives for diverse, appropriate, affordable, and safe housing.	Everyone	• Improved housing stability, employment and related skills, health, and healthy behaviours.<br>• Reduced stress, increased savings, feelings of security, homelessness.<br>• People have paid off 'street debt' and other loans (e.g., payday lenders).<br>• Housing choice has greatly reduced re-housing.	30
